# A layered stacking ensemble approach for missing data imputation

**DOI:** 10.1371/journal.pone.0359843

**Published:** 2026-10-12

**Authors:** Muhammad Asif, Klairung Samart

**Affiliations:** 1 Department of Economics, Faculty of Management and Social Sciences, Lasbela University of Agriculture, Water and Marine Sciences, Uthal, Balochistan, Pakistan; 2 College of Digital Science, Prince of Songkla University, Hat Yai, Songkhla, Thailand; 3 Research Center in Mathematics and Statistics with Applications, Prince of Songkla University, Hat Yai, Songkhla, Thailand; 4 Division of Computational Science, Faculty of Science, Prince of Songkla University, Hat Yai, Songkhla, Thailand; The University of Texas, MD Anderson Cancer Center, UNITED STATES OF AMERICA

## Abstract

Missing data is a challenging issue in data analysis. Researchers often address the issue by simply removing incomplete cases from the analysis. However, this approach reduces the sample size and can undermine the accuracy of statistical estimates. Alternatively, imputation techniques can be used to replace missing values with plausible estimates, resulting in a more complete dataset and more reliable estimates. In this study, we propose a new imputation method, which we call Layered Stacking. The method is designed to improve the estimation of continuous missing values. To evaluate its effectiveness, comprehensive simulation studies were carried out with four distinct data generating processes using datasets generated under the missing at random mechanism, with sample sizes of 500, 1,000, 5,000 and 10,000, and missingness rates of 10, 20, 30 and 40%. The proposed method is compared with five widely used imputation techniques: k-nearest neighbors, support vector regression, decision tree, random forest and extreme gradient boosting. Evaluation metrics include mean squared imputation error (MSIE) and mean absolute percentage imputation error (MAPIE) along with standard deviation (SD) and 95% confidence interval (CI) across replications. Two real-world datasets have also been considered to assess the performance of the method. The proposed method generally achieved lower MSIE than the benchmark models, particularly for larger datasets.

## Introduction

Data quality is a critical factor for data scientists and researchers in the fields of data science and analytics. Key preliminary steps in data analysis include pre-processing, which involves data preparation, cleaning, integration, transformation, reduction, and discretization. Each of these steps plays an important role in ensuring the overall quality and reliability of the data [1]. The final results of the analysis are generated by statistical and machine learning (ML) algorithms whose performance is based on factors such as feature selection, algorithm choice, sampling methods, training, testing, and validation of datasets. Following these conditions, a substantial obstacle encountered by data scientists or researchers is missing data, which is thought to be one of the most important problems in data analysis [2–4]. Most statistical and ML methods exhibit insufficient resilience when processing incomplete datasets. In the ideal scenario, missing data is prevented, but in many real-world situations, this is not always possible [5,6]. Data may be missing for a variety of reasons. For instance, respondents might decline to answer certain questions, lack the required knowledge, or deliberately skip them. In addition, missing data can result from equipment failures or incidents such as the death of patients [7–9]. Regardless of the reasons, it is important to accept that most statistical and ML algorithms fail to handle missing data effectively [5], which can seriously affect the performances of these algorithms [10,11]. When data values are absent, an element of ambiguity is introduced into the analysis. This uncertainty can negatively impact the properties of statistical estimators, reducing statistical power and potentially leading to inaccurate or invalid conclusions. Effectively handling missing values is both essential and complex, requiring a thorough analysis of all data instances to identify missing patterns within the dataset. In addition, it requires a deep understanding of various imputation techniques.

Missing data mechanisms play a crucial role in determining a suitable imputation approach. Missing data mechanisms allow researchers to assess whether missing data is systematically related to observed variables, thus guiding appropriate imputation strategies [12]. These insights elaborate the nature and underlying causes of missingness patterns within the dataset [13,14]. Referring to Rubin’s framework [15], missing data patterns can be classified into three categories [16]: missing completely at random (MCAR), missing at random (MAR) and missing not at random (MNAR). Under the MCAR mechanism, the occurrence of missing data is entirely independent of any data values, whether observed or unobserved. MAR occurs when the likelihood of a missing data point is associated with the observed data, but not with the value of the missing data itself. Data is considered MNAR when the likelihood of a missing data point depends on the missing value itself [17]. These classifications help us understand how data is missing and guide our approach to handling it.

Established approaches to handling missing data are generally classified into two main categories: deletion and imputation. Deletion methods are the simplest and most convenient ways that involve omitting cases or variables with missing values, potentially leading to a reduced sample size and the loss of valuable information [17]. A more effective way to handle missing values is by imputing them using an appropriate technique. Imputation aims to estimate or substitute missing values with plausible values, thereby providing a more complete dataset for analysis. Imputation techniques are increasingly popular because they are both practical and reliable. They are practical since they can be applied after data collection, and reliable because they take advantage of the patterns in the missing data [18]. Proper missing data management preserves valuable information, enabling the analysis of as much relevant information as possible. The choice of deletion or imputation depends on the research objectives, the characteristics of the dataset, and the potential effect on the validity of the statistical analysis. Many imputation strategies, using both statistical and ML approaches, have been discussed in the literature. Statistical methods impute missing data by using statistical properties of data. Common approaches include multiple imputation, regression imputation, or mean substitution. ML-based imputation methods depend on building predictive models to estimate missing values using the available information within the dataset. Such approaches involve traditional ML models, such as k-nearest neighbor (kNN), decision tree (DT), or linear regression (LR) [18]. With the rise of complex data structures such as non-linear, hierarchical or longitudinal models, classical methods are often insufficient, requiring the need for innovative approaches to effectively address the analytical demands of modern datasets [19,20].

Recent research has demonstrated the critical role of imputation techniques in improving the performance of predictive models across various domains. In a study by Aljrees [21], various preprocessing techniques, including deletion, kNN imputation, and PCA based imputation, were used to address missing values in the dataset. The author evaluated several state-of-the-art ML models along with his proposed multi-model ensemble learning technique on the imputed data to assess predictive performance for early-stage cervical cancer detection. Experimental results demonstrated that the ensemble method significantly outperformed baseline models, achieving scores of 0.9941 for accuracy, 0.98 for precision, 0.96 for recall, and 0.97 for *F*_1_. Alnowaiser [22], continuing the work of Aljrees, used deletion and kNN imputation to address missing data, and subsequently evaluated classical ML models alongside a proposed tri-ensemble method for early-stage diabetes prediction. The results demonstrated the better performance of the tri-ensemble approach, achieving an accuracy of 90.03%, a precision of 93.46%, a recall of 93.21%, and an *F*_1_ score of 93.33%, outperforming all baseline methods across all metrics.

Khan et al. [18] conducted a study in which they introduced a novel imputation technique called the ranked k-nearest neighbors (RkNN) method as an improvement over the traditional kNN algorithm. Both methods were applied to simulated and real-world datasets, with missing values artificially introduced under MCAR and MAR mechanisms. The performances of the imputation methods were evaluated using the Mean Square Imputation Error (MSIE) and Mean Absolute Imputation Error (MAIE) metrics. The proposed RkNN method outperformed the traditional kNN algorithm in terms of imputation accuracy. Another study by Thongsri and Samart [23] conducted a comparative analysis, using list-wise deletion and single imputation techniques against their proposed approach. Their proposed method involved a combination of stochastic regression (SR) and random forest (RF), utilizing equivalent weight. This comparison was carried out through a simulation study. The results clearly demonstrated the superior performance of the proposed method over the alternative approaches. Continuing their prior research [24], the authors extended their proposed imputation method by integrating three distinct imputation techniques: kNN, SR, and RF, utilizing equivalent weights (kSREW). They conducted a thorough comparative analysis, evaluating kSREW against several other established imputation methods, including HD, kNN, SR, predictive mean matching imputation (PMM), RF, stochastic regression random forest with equivalent weight imputation (SREW), and k-nearest random forest with equivalent weight imputation (kREW). The assessment involved both simulation and real-world data. The proposed combination of three methods with kSREW outperformed the aforementioned imputation techniques.

The literature shows that single ML imputation techniques often exhibit limited effectiveness in accurately predicting missing values. While recent advancements have demonstrated the potential of ML approaches, including ensemble models, in improving the prediction of study variable outcomes, comprehensive evaluations of their combined impact on imputation accuracy remain limited. Furthermore, there is a noticeable lack of research into the integration of stacking ensemble imputation with classical ML methods to enhance the prediction of missing values. This identified gap highlights the motivation and necessity of the present study, wherein we introduce a novel stacking ensemble imputation approach aimed at improving the accuracy of missing value estimation.

The proposed method utilizes the predictive strengths of diverse regression algorithms, structured into multiple layers, to effectively use the available data for imputation. By combining the outputs of base learners through a meta-learner, the model captures complex patterns that may be overlooked by individual algorithms. To evaluate its effectiveness, the layered stacking ensemble is compared with traditional ML techniques using regression-based imputation.

This paper is organized into the following sections, each focusing on a specific aspect of the research. Section 2 presents a detailed explanation of each imputation method, including the proposed approach, along with a description of the evaluation metrics and the simulation process. Section 3 presents the results of the simulation study conducted to evaluate the performance of the proposed method. Finally, Section 4 summarizes the key findings and conclusions, highlighting both the strengths and limitations of the proposed approach and offering recommendations for future research in this area.

## Methodology

In this study, we consider single and ensemble ML imputation methods in terms of regression, in which $Y_{i}$ is a non-linear response variable where *i* = 1, 2, ....., *n* with *m* missing values, and *X*_1*i*_ and *X*_2*i*_ are independent variables. The procedure for supervised learning is to train a model to predict unknown outcomes from known input and output data, where the input data is associated with known labels or target values. Regression imputation methods are then used to predict the unknown numerical output values. The supervised ML methods used in this study are as follows.

## Imputation methods

### k-nearest neighbor (kNN)

kNN imputation is a widely used non-parametric technique for addressing missing data in ML and data analysis. This method replaces missing values with the average of the values from the ‘*k*’ most similar instances in the dataset, where ‘*k*’ is a parameter specified by the user [7].

Following are the steps used for implementing kNN.

1. Each data point in the dataset is defined both by features (independent variables) and the corresponding target variable (dependent variable).
2. The value of *k*, representing the number of neighbors to choose, is a hyperparameter that can be adjusted.
3. When predicting the target value for a new data point, the algorithm identifies the nearest *k* neighbors using a distance metric (e.g., Euclidean distance).
4. The target value for the unknown data point is obtained by taking the average of the values from its nearest *k* neighbors.

In the literature, a variety of similarity measures have been proposed to calculate distances between donors and recipients. Among these, Euclidean distance is one of the most widely used metrics, which we explain below.

Let there be a data matrix *X* of dimensions ${\mathbb{R}}^{n\times p}$, where each element $x_{ij}$ represents the value of the $j^{th}$ variable for the $i^{th}$ observation. Correspondingly, let *V* be a missingness indicator matrix of the same dimensions, where $v_{ij}=1$ if $x_{ij}$ is observed and $v_{ij}=0$ otherwise. The distance between the $i^{th}$ and $j^{th}$ observations, denoted as $d(i^{th},j^{th})$ can be calculated using the Euclidean distance formula, applied only to variables observed in both observations.


$$\begin{array}{c} d({𝐱}_{i},{𝐱}_{j})=\sqrt{\frac{1}{T_{ij}}\sum_{r=1}^{q}(x_{i,r}-x_{j,r}{)}^{2}\cdot I(v_{i,r}=1)\cdot I(v_{j,r}=1)} \end{array}$$ (1)

where $T_{ij}$ denotes the sum of indicator variables, while $v_{ir}=1$ and $v_{jr}=1$ for each variable *r* = 1,2,3...,*q*. It represents the number of valid components for which both vectors $x_{i}$ and $x_{j}$ have available values. The indicator function *I*(*v*) equals 1 when the condition *v* is true, and 0 otherwise. Therefore, the use of these indicator functions ensures that only the components present in both $x_{i}$ and $x_{j}$ contribute to the calculation of the distance metric [18].

### Support vector regression (SVR)

SVR is a supervised learning model with associated learning algorithms designed for regression analysis. Developed by Müller et al. [25], SVR is rooted in the concept of SVM. This data-driven model has been widely adopted in ML for regression tasks and is particularly valuable when the data is not linearly separable. It supports applicability in both classification and regression tasks.

The primary objective of SVR is to predict a relationship between the input vector $x\in {\mathbb{R}}^{n}$ and the output variable y∈ℝ. During the training phase, SVR learns by identifying the optimal weights and biases that best fit input-output pairs provided in the training dataset [26]. Subsequently, the trained SVR model can estimate outputs for new unknown values, utilizing the established weights, biases, and knowledge acquired during training [27,28].

### Decision tree (DT) regression

DT builds a regression model by repeatedly dividing the dataset into smaller subsets while developing an associated tree structure. The data is divided through decision nodes, where each node has two or more branches representing different attribute values. As the tree grows, leaf nodes emerge, signifying the ultimate decisions on the numerical target. The very first node at the top, known as the root node, corresponds to the most significant predictor in the regression model [26].

- **DT algorithm:** The primary algorithm for constructing decision trees is known as iterative dichotomiser 3 (ID3), originally developed by Quinlan, J. R [29]. It follows a top-down, greedy search pattern within the space of potential branches without incorporating backtracking. For regression tasks, the ID3 algorithm can be adapted by replacing information gain (IG) with variance reduction (VR) as the criterion for node splitting. This enables the construction of decision trees optimized for regression analysis.

The formula for variance reduction is given as:


$$\begin{array}{c} VR=\text{var(root node)}-\sum_{i}w_{i}\text{var(child nodes)} \end{array}$$ (2)

where $w_{i}=\frac{n_{i}}{n}$, is the weight of the $i^{th}$ child node relative to the parent node, $n_{i}$ is the number of observations in the $i^{th}$ child node and *n* is the total number of observations in the parent node.

### Random forest (RF) regression

RF regression is an extension of the RF algorithm designed to address regression problems. While classical RF is commonly known for its use in classification tasks, RF regression is structured for predicting continuous numerical values. It belongs to the family of ensemble methods, where multiple models are combined to create a more robust and accurate predictor [27]. The RF regression algorithm operates as follows:

a) *k* data points are randomly selected from the training set.
b) Decision trees are generated based on the *k* selected data points.
c) The number of trees, denoted as *N*, is specified and steps (a) and (b) are repeated for each tree.
d) The ensemble of *N* trees is utilized to predict the numerical value for a new data point. The final result is the average of predictions from the *N* trees.

### Extreme gradient boosting (XGB)

XGB, proposed by Chen et al. [30], is an advanced ML algorithm designed for efficiency, speed, and better performance. It extends the classical gradient boosting by including regularization terms in the objective function, improving generalization and preventing overfitting [30,31]. XGB works as follows:

- The process begins by training an initial DT model, which typically predicts the average value of the target variable in regression tasks.
- The residual errors (differences between actual and predicted values) are then computed.
- A new tree is trained on these residuals to correct the errors of the previous tree.
- This procedure is repeated iteratively, with each new tree attempting to minimize the loss function (e.g., squared error for regression) based on the gradient of the residuals.
- The final output is obtained by summing the predictions from all individual trees.

Regularization techniques and learning rate adjustments are used to prevent overfitting and improve generalization. The model is mathematically expressed as:


$$\begin{array}{c} \hat{y_{i}}=\sum_{m=1}^{M}f_{m}(x_{i}) \end{array}$$ (3)

where $\hat{y_{i}}$ is the final predicted value for the $i^{th}$ data point, *M* is the number of trees in the combination and $f_{m}(x_{i})$ represents the prediction of the $m^{th}$ tree for the $i^{th}$ data point.

The objective function in XGB comprises two parts: a loss function and a regularization term.


$$\begin{array}{c} obj(\theta )=\sum_{i}^{n}l(y_{i},\hat{y_{i}})+\sum_{m}^{M}\Omega (f_{m}) \end{array}$$ (4)

$l(y_{i},\hat{y_{i}})$ is the loss function which computes the difference between the true value $y_{i}$ and the predicted value $\hat{y_{i}}$. $\Omega (f_{m})$ is the regularization term that helps to prevent the model from becoming too complex. When deciding how to split the nodes in the tree, we compute the IG for every possible split. The IG for a split is calculated as:


$$\begin{array}{c} Gain=\frac{1}{2}\left[\frac{G_{L}^{2}}{H_{L}+\lambda }+\frac{G_{R}^{2}}{H_{R}+\lambda }-\frac{(G_{L}+G_{R}{)}^{2}}{H_{L}+H_{R}+\lambda }\right]-\gamma \end{array}$$ (5)

- $G_{L}$ is the sum of first-order derivatives (gradients) of the loss function for all data points in the left child node, and $G_{R}$ the sum of gradients for all data points in the right child node.
- $H_{L}$ is the sum of second order derivatives (Hessians) of the loss function for all data points in the left child node, and $H_{R}$ is the sum of Hessians in the right child node.
- γ penalizes the square of the leaf weights in the tree.

XGB evaluates the IG for every possible split at each node and chooses the split that gives the highest gain, thereby effectively minimizing errors and enhancing the performance of the model.

### Layered stacking

Ensemble learning is a powerful technique that combines multiple individual models to form a more accurate and efficient predictive model [32]. Stacking is an ensemble method in which the predicted results of each imputation method are combined for each sample input and aggregated into a new feature vector. This vector is then passed to a meta-learner, which generates the final predicted results.

In this study, we introduce a new variation of the stacked model we call “Layered Stacking.” The proposed model is structured in multiple layers, leveraging the strengths of five different learning models. The first layer uses predictions from two classical ML models, KNN and SVR, as input to train the tree-based models DT, RF, and XGB.

In the second layer, predictions from these tree-based models are used to train the meta-learners (i.e., second-degree polynomial regression, ridge regression, or linear regression), in the last layer, the meta-learner generates the final imputed values. This stacked approach allows the meta-learner to effectively learn how to combine the outputs from the base models. By adaptively assigning weights to their predictions, the stacked ensemble achieves improved performances compared with any individual imputation method alone [33]. Using this stacking technique, the multilayer ensemble structure shows various advantages. Statistically, the ensemble layer reduces the risk of incorrect predictions by combining predictions from different layer combinations. The diversity of the models ensures an improved approximation of the prediction between the input and output values, which significantly eliminates the chance of determining a local optimum [34]. The architecture of the proposed model is illustrated in Fig 1.

**Fig 1:**
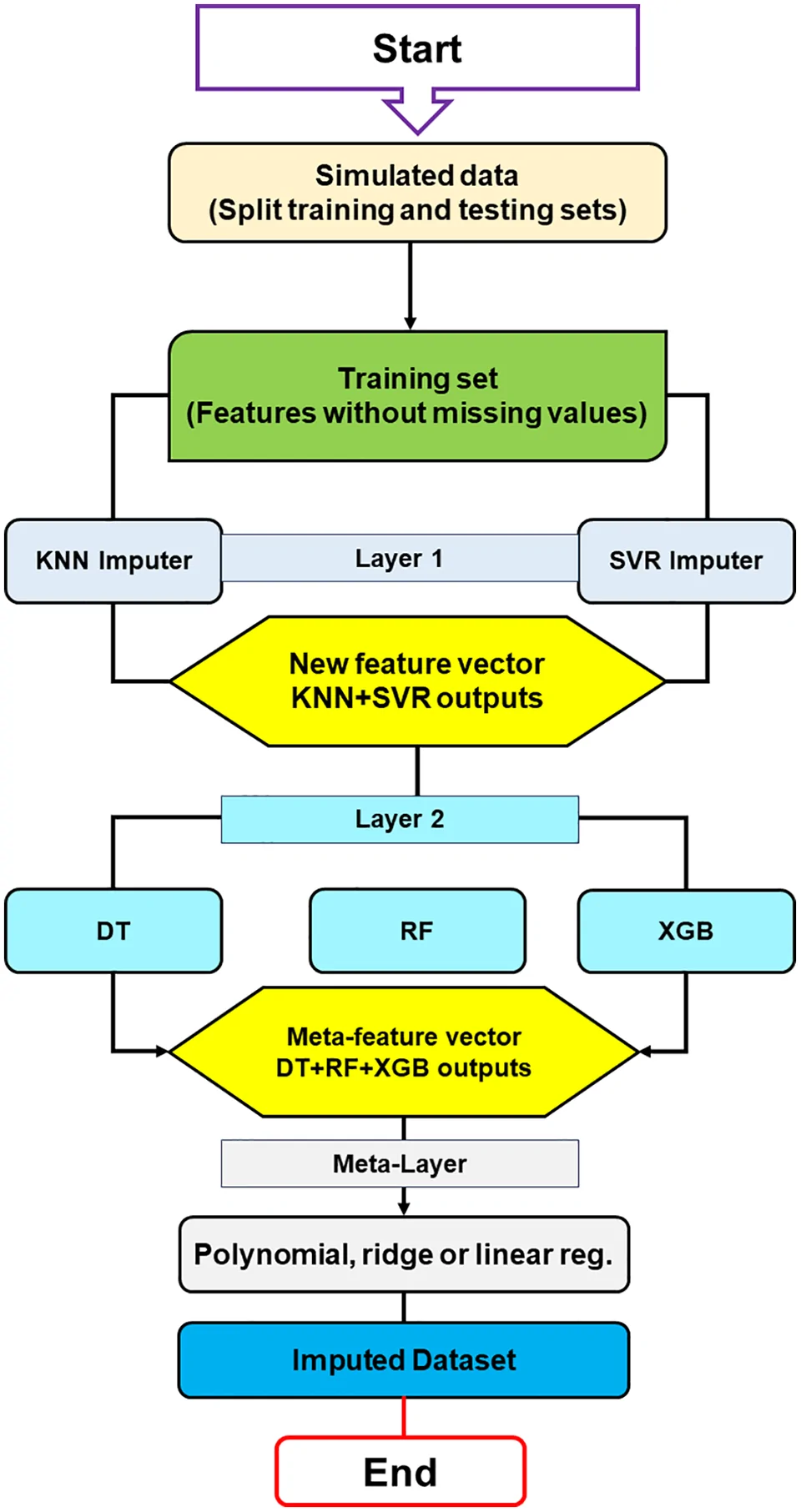
Layer architecture of the proposed method.

### Performance evaluation metrics

To ensure the reliability of the imputed data, it is important to assess how closely the imputed values match the true values. Two widely used evaluation metrics for this purpose are the mean square imputation error (MSIE) and the mean absolute percentage imputation error (MAPIE). These metrics are calculated by comparing the imputed values with the actual (true) values.


$$\begin{array}{c} \text{MSIE}=\frac{1}{n}\sum_{i=1}^{n}{\left(y_{i}-{\hat{y}}_{i}\right)}^{2} \end{array}$$ (6)

and


$$\begin{array}{c} \text{MAPIE}=\frac{100}{n}\sum_{i=1}^{n}\left|\frac{y_{i}-{\hat{y}}_{i}}{y_{i}}\right| \end{array}$$ (7)

In Eq (6) and (7), *n* is the total number of missing values, $y_{i}$ is the original value, and $\hat{y_{i}}$ is the value used to replace the missing data. MSIE and MAPIE measure the average squared difference and the absolute percentage difference between the true and imputed values, respectively. Both metrics are always non-negative, with a value of zero indicating perfect imputation accuracy.

### Simulation study

We carried out a comprehensive simulation study to compare the performances of the ML imputation methods using the model proposed and discussed in the previous section. Sample sizes of 500, 1000, 5000 and 10000 were randomly generated. The values of the independent variables were sampled from normal distributions, $X_{j}\sim N(0,1)$ where *j* = 1,2 represents the two independent variables included in the models. The corresponding values of the study variables $\left(Y_{i}\right)$ were then generated based on its predetermined nonlinear relationship with predictors *X*_1*i*_ and *X*_2*i*_, specified by equations


$$\begin{array}{c} Y=0.3+0.5X_{1}+0.7{X_{2}}^{2} \end{array}$$ (8)

$$\begin{array}{c} Y=0.2+0.5\log(|X_{1}|+1)+0.4\log(|X_{2}|+1)+0.7X_{2}^{2}+\epsilon_{i} \end{array}$$ (9)

where


$$\begin{array}{c} \epsilon_{i}=𝒩(0,0.2)+𝒩(0,0.3)\;\mathrm{sign}(X_{1}X_{2}) \end{array}$$

$$\begin{array}{c} Y=1+0.6X_{1}-0.4X_{2}+0.8{X_{1}}^{3}+0.5{X_{2}}^{4}+\epsilon_{i} \end{array}$$ (10)

where


$$\begin{array}{c} \epsilon_{i}=𝒩(0,0.3{X_{1}}^{2}) \end{array}$$

$$\begin{array}{c} Y=1+0.3X_{1}+0.5X_{2}+0.6{X_{1}}^{2}+0.8X_{1}X_{2}+\epsilon_{i} \end{array}$$ (11)

where


$$\begin{array}{c} \epsilon_{i}=𝒩(0,1) \end{array}$$

Missingness in the study variable $Y_{i}$ was artificially introduced under the MAR mechanism for each sample size. The original simulated datasets contained no missing values. We considered missingness rates of 10, 20, 30, and 40%. The missing values of the study variable $Y_{i}$ were then predicted using ML imputation methods. MSIE and MAPIE, together with their corresponding SDs and 95% CIs were calculated. Because imputation methods measure the accuracy of a continuous variable, the MSIE and MAPIE are good choices. The simulation process was repeated 1,000 times and all simulations were performed using the RStudio software. The flowchart Fig 2 explains the methodology of the study.

**Fig 2:**
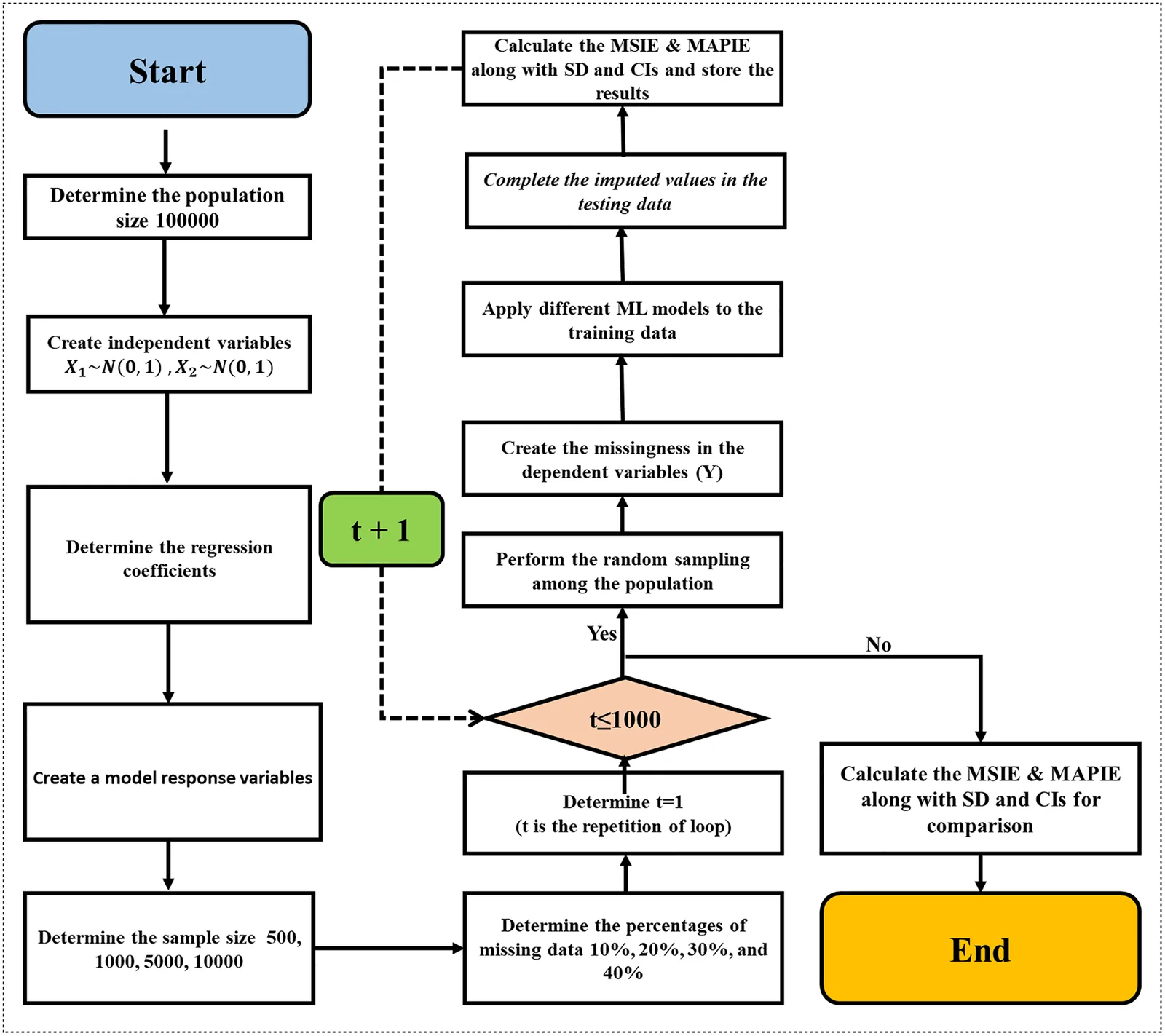
Flowchart of the simulation methodology.

## Results and discussion

In this section we describe the performance evaluation of various ML imputation methods, including kNN, SVR, DT, RF, XGB, and stacking. Among these methods, kNN, SVR, and DT are single ML models, whereas RF, XGB, and stacking are ensemble learning methods. The single ML imputation approach replaces each missing value with a single estimated value generated based on the training data. Ensemble methods combine predictions from multiple single models to construct a more robust imputation model, thereby better capturing the uncertainty and complexity associated with determining the appropriate values to impute [35]. ML models are trained on observed data containing features without missing values, while attributes associated with missing values are used to test the performance of the model [16]. The stacking approach introduced in this study is our proposed Layered Stacking method. The idea behind the proposed multi-layered model architecture is inspired by the design principles of artificial neural networks (ANNs), particularly their sequential processing through input, hidden, and output layers, which allows the capture of complex patterns and dependencies within the data. However, unlike traditional ANNs, the proposed model does not include a hidden layer. We have used second-degree polynomial regression for the first and second DGPs, ridge regression for third DGP, and linear regression for the fourth DGP as the meta-learners. The choice of meta-learner is further supported from a study conducted by Wang and Lu [35], which states that “the selection of meta learners is flexible, with no strict constraints” and may appropriately be guided by the nature of the data and the characteristics of the base learners employed. In addition, to prevent information leakage in the stacking framework, 5-fold out-of-fold (OOF) predictions were used to generate the training data for the meta-learner in the fourth DGP. By using these approaches, the proposed method effectively minimizes both MSIE and MAPIE, thereby enhancing imputation accuracy, especially in scenarios involving large sample sizes. Tables 1–8 show the performances of the imputation methods from first and second DGPs, and the Tables 9–16 describe the performances of the imputation methods from third and fourth DGPs in terms of regression accuracy. The impacts of missing rates and sample sizes on the imputation methods were studied. Specifically, each dataset was generated with a MAR mechanism at four levels of missingness. In particular, the imputation performance of the proposed method was compared with the performances of the component imputation methods, kNN, SVR, DT, RF, and XGB. The results demonstrate that the proposed method generally performed more favorably than other imputation approaches, and had superior performance especially on larger sample sizes (5,000 and 10,000), where it achieved lower MSIE values for almost all levels of missingness, highlighting its effectiveness and scalability with increasing data volume under MAR missningness mechanism. However, the evaluation based on MAPIE revealed that no single method consistently outperformed the others under all conditions. Nevertheless, the proposed method consistently achieved the second-best performance in terms of MAPIE in the majority of experimental scenarios. Although the numerical improvements of the proposed stacking ensemble method with some sample sizes and missing percentages appear equivalent or lower in scale than base models particularly RF, but the gains of proposed method are consistent across all 1,000 simulation replications. It is important to note that in regression problems involving large datasets, even marginal improvements in predictive accuracy can result in practically meaningful reductions in imputation error [18]. To assess whether the observed differences between methods are significant, Friedman tests were conducted across all simulation replications for both MSIE and MAPIE metrics. The results were statistically significant in all scenarios (*p* < 0.001), supporting that the performance differences among the imputation methods are not attributable to chance. The better efficacy of the proposed Layered Stacking ensemble approach suggests that optimizing predictions from several base learners across several layers can lead to more accurate imputation results, especially when processing large datasets under the MAR missingness mechanism.

**Table 1:** Average MSIE and MAPIE with standard deviations (SD) and 95% confidence intervals (CI) for first DGP obtained from different imputation methods for *n* = 500.

Missing (%)	Method	MSIE	SD	95% CI	MAPIE	SD	95% CI
10%	KNN	0.1768	0.1932	(0.1649, 0.1888)	7.2572	1.3159	(7.1757, 7.3388)
	SVR	0.1367	0.2260	(0.1228, 0.1508)	11.4948	1.4715	(11.4036, 11.5860)
	DT	0.1651	0.2153	(0.1518, 0.1785)	15.9121	2.0188	(15.7871, 16.0373)
	RF	**0.1188**	0.1972	(0.1066, 0.1311)	3.2785	0.9682	(3.2186, 3.3386)
	XGB	0.1346	0.2196	(0.1211, 0.1483)	3.2073	0.9969	(3.1455, 3.2691)
	Stacking	0.1223	0.2075	(0.1095, 0.1352)	**3.0644**	0.9846	(3.0034, 3.1254)
20%	KNN	0.2443	0.1833	(0.2330, 0.2557)	8.8846	1.3123	(8.8033, 8.9659)
	SVR	0.1900	0.2114	(0.1769, 0.2031)	12.0029	1.3541	(11.9190, 12.0869)
	DT	0.2203	0.2078	(0.2075, 0.2332)	16.5392	1.7229	(16.4325, 16.6460)
	RF	**0.1738**	0.1865	(0.1623, 0.1854)	4.1158	0.9532	(4.0567, 4.1749)
	XGB	0.1900	0.2073	(0.1772, 0.2029)	**3.6728**	0.9515	(3.6139, 3.7319)
	Stacking	0.1755	0.1970	(0.1633, 0.1877)	3.7081	0.9462	(3.6495, 3.7668)
30%	KNN	0.3505	0.2019	(0.3380, 0.3630)	12.0746	1.9673	(11.9527, 12.1966)
	SVR	0.2602	0.2142	(0.2470, 0.2735)	12.8849	1.2839	(12.8054, 12.9645)
	DT	0.2966	0.2156	(0.2833, 0.3100)	17.5142	1.9159	(17.3955, 17.6330)
	RF	0.2564	0.2025	(0.2439, 0.2690)	5.8225	1.3562	(5.7385, 5.9066)
	XGB	0.2670	0.2161	(0.2537, 0.2805)	**4.6880**	1.2558	(4.6102, 4.7659)
	Stacking	**0.2516**	0.2092	(0.2387, 0.2646)	5.0274	1.3057	(4.9465, 5.1084)
40%	KNN	0.8063	0.4052	(0.7813, 0.8315)	25.5849	8.3947	(25.0646, 26.1053)
	SVR	0.5143	0.2924	(0.4963, 0.5325)	17.1790	4.1234	(16.9235, 17.4347)
	DT	0.5262	0.2752	(0.5092, 0.5433)	21.0193	3.5454	(20.7996, 21.2391)
	RF	0.5593	0.3083	(0.5402, 0.5785)	13.4768	5.1897	(13.1552, 13.7985)
	XGB	0.5085	0.2764	(0.4915, 0.5257)	**10.0192**	4.5077	(9.7399, 10.2987)
	Stacking	**0.4791**	0.2632	(0.4628, 0.4955)	11.3795	4.9183	(11.0747, 11.6843)

Note: SD = Standard deviation; CI = 95% Confidence interval based on 1,000 simulation replications.

**Table 2:** Average MSIE and MAPIE with standard deviations (SD) and 95% confidence intervals (CI) for first DGP obtained from different imputation methods for *n* = 1000.

Missing (%)	Method	MSIE	SD	95% CI	MAPIE	SD	95% CI
10%	KNN	0.1141	0.1498	(0.1049, 0.1234)	5.0015	0.7109	(4.9574, 5.0456)
	SVR	0.1022	0.1950	(0.0902, 0.1144)	11.3569	1.0849	(11.2896, 11.4241)
	DT	0.1315	0.1884	(0.1198, 0.1432)	15.5803	1.4220	(15.4922, 16.0373)
	RF	**0.0828**	0.1631	(0.0727, 0.0929)	**2.1035**	0.5258	(2.0709, 2.1360)
	XGB	0.0962	0.1820	(0.0850, 0.1075)	2.4142	0.5592	(2.3796, 2.4489)
	Stacking	0.0844	0.1685	(0.0740, 0.0949)	2.1255	0.5475	(2.0916, 2.1595)
20%	KNN	0.1469	0.1057	(0.1404, 0.1535)	6.0822	0.6887	(6.0396, 6.1250)
	SVR	0.1268	0.1370	(0.1183, 0.1353)	11.5766	0.9125	(11.5201, 11.6332)
	DT	0.1586	0.1369	(0.1502, 0.1672)	16.0685	1.2134	(15.9933, 16.1437)
	RF	**0.1069**	0.1156	(0.0998, 0.1141)	2.5843	0.4884	(2.5541, 2.6146)
	XGB	0.1233	0.1333	(0.1151, 0.1316)	2.6549	0.5072	(2.6239, 2.6859)
	Stacking	0.1094	0.1229	(0.1018, 0.1170)	**2.5428**	0.5004	(2.5114, 2.5743)
30%	KNN	0.2101	0.1126	(0.2032, 0.2171)	8.2077	0.9770	(8.1471, 8.2682)
	SVR	0.1734	0.1324	(0.1652, 0.1816)	12.1173	0.8840	(12.0625, 12.1721)
	DT	0.2068	0.1333	(0.1986, 0.2151)	16.5568	1.2861	(16.4771, 16.6366)
	RF	**0.1562**	0.1177	(0.1489, 0.1635)	3.5394	0.6264	(3.5006, 3.5783)
	XGB	0.1717	0.1306	(0.1637, 0.1799)	**3.1259**	0.5762	(3.0901, 3.1616)
	Stacking	0.1568	0.1221	(0.1493, 0.1644)	3.3201	0.6518	(3.2797, 3.3605)
40%	KNN	0.5112	0.2577	(0.4953, 0.5272)	17.2635	5.4445	(16.9260, 17.6010)
	SVR	0.3504	0.1730	(0.3317, 0.3531)	14.4066	2.1367	(14.2743, 14.5391)
	DT	0.3751	0.1732	(0.3644, 0.3859)	18.3990	1.9904	(18.2756, 18.5224)
	RF	0.3654	0.1953	(0.3533, 0.3775)	8.3370	3.1714	(8.1405, 8.5336)
	XGB	0.3521	0.1803	(0.3409, 0.3633)	7.5113	2.4351	(7.3181, 7.7045)
	Stacking	**0.3424**	0.1910	(0.3386, 0.3623)	**5.9501**	3.1165	(5.7991, 6.1010)

Note: SD = Standard deviation; CI = 95% Confidence interval based on 1,000 simulation replications.

**Table 3:** Average MSIE and MAPIE with standard deviations (SD) and 95% confidence intervals (CI) for first DGP obtained from different imputation methods for *n* = 5000.

Missing (%)	Method	MSIE	SD	95% CI	MAPIE	SD	95% CI
10%	KNN	0.0320	0.0282	(0.0302, 0.0337)	2.0862	0.1485	(2.0770, 2.0954)
	SVR	0.0391	0.0481	(0.0361, 0.0421)	11.2773	0.4498	(11.2494, 11.3052)
	DT	0.0734	0.0455	(0.0706, 0.0762)	15.3075	0.5935	(15.2707, 15.3443)
	RF	0.0233	0.0356	(0.0210, 0.0254)	**0.7276**	0.1210	(0.7201, 0.7351)
	XGB	0.0300	0.0414	(0.0274, 0.0325)	1.7111	0.1395	(1.7025, 1.7198)
	Stacking	**0.0233**	0.0357	(0.0211, 0.0255)	0.8593	0.1549	(0.8497, 0.8689)
20%	KNN	0.0409	0.0229	(0.0395, 0.0423)	2.5064	0.1477	(2.4972, 2.5156)
	SVR	0.0468	0.0357	(0.0445, 0.0490)	11.3783	0.4244	(11.3520, 11.4046)
	DT	0.0813	0.0343	(0.0792, 0.0835)	15.7268	0.5477	(15.6929, 15.7607)
	RF	0.0302	0.0274	(0.0285, 0.0319)	**0.8717**	0.1275	(0.8638, 0.8796)
	XGB	0.0375	0.0319	(0.0355, 0.0395)	1.7633	0.1397	(1.7546, 1.7719)
	Stacking	**0.0301**	0.0276	(0.0280, 0.0328)	1.0109	0.1719	(1.0003, 1.0216)
30%	KNN	0.0629	0.0259	(0.0613, 0.0645)	3.3955	0.2062	(3.3827, 3.4083)
	SVR	0.0678	0.0378	(0.0654, 0.0701)	11.5162	0.3997	(11.4915, 11.5410)
	DT	0.1028	0.0367	(0.1005, 0.1051)	15.9394	0.5970	(15.9024, 15.9764)
	RF	0.0490	0.0302	(0.0471, 0.0508)	**1.1862**	0.1561	(1.1765, 1.1959)
	XGB	0.0584	0.0348	(0.0562, 0.0605)	1.8962	0.1708	(1.8856, 1.9068)
	Stacking	**0.0488**	0.0303	(0.0469, 0.0507)	1.3491	0.2097	(1.3361, 1.3621)
40%	KNN	0.1566	0.0475	(0.1537, 0.1596)	6.8228	0.7972	(6.7734, 6.8722)
	SVR	0.1406	0.0498	(0.1375, 0.1437)	12.1127	0.4319	(12.0859, 12.1394)
	DT	0.1703	0.0498	(0.1672, 0.1733)	16.2538	0.6459	(16.2138, 16.2938)
	RF	0.1216	0.0457	(0.1187, 0.1244)	2.5719	0.4201	(2.5459, 2.5979)
	XGB	0.1331	0.0493	(0.1300, 0.1361)	**2.5229**	0.3342	(2.5022, 2.5436)
	Stacking	**0.1212**	0.0451	(0.1184, 0.1241)	2.8545	0.5968	(2.8176, 2.8915)

Note: SD = Standard deviation; CI = 95% Confidence interval based on 1,000 simulation replications.

**Table 4:** Average MSIE and MAPIE with standard deviations (SD) and 95% confidence intervals (CI) for first DGP obtained from different imputation methods for *n* = 10,000.

Missing (%)	Method	MSIE	SD	95% CI	MAPIE	SD	95% CI
10%	KNN	0.0189	0.0147	(0.0180, 0.0198)	1.4409	0.0751	(1.4362, 1.4456)
	SVR	0.0292	0.0269	(0.0275, 0.0308)	11.3555	0.3171	(11.3359, 11.3752)
	DT	0.0634	0.0236	(0.0620, 0.0649)	15.2779	0.4433	(15.2504, 15.3053)
	RF	0.0139	0.0185	(0.0128, 0.0151)	**0.4610**	0.0679	(0.4568, 0.4652)
	XGB	0.0188	0.0209	(0.0175, 0.0201)	1.6225	0.0859	(1.6172, 1.6278)
	Stacking	**0.0139**	0.0182	(0.0123, 0.0150)	0.5768	0.1139	(0.5688, 0.5829)
20%	KNN	0.0237	0.0127	(0.0229, 0.0245)	1.7247	0.0715	(1.7203, 1.7292)
	SVR	0.0333	0.0225	(0.0319, 0.0347)	11.4245	0.2828	(11.4070, 11.4421)
	DT	0.0680	0.0201	(0.0668, 0.0693)	15.6615	0.4002	(15.6367, 15.6863)
	RF	0.0177	0.0161	(0.0167, 0.0187)	0.6804	0.0683	(0.6725, 0.6878)
	XGB	0.0231	0.0183	(0.0220, 0.0243)	1.6661	0.0911	(1.6605, 1.6718)
	Stacking	**0.0177**	0.0159	(0.0167, 0.0187)	**0.6723**	0.1224	(0.6717, 0.6861)
30%	KNN	0.0372	0.0138	(0.0363, 0.0380)	2.3217	0.1056	(2.3151, 2.3282)
	SVR	0.0472	0.0219	(0.0458, 0.0485)	11.4966	0.2910	(11.4785, 11.5146)
	DT	0.0819	0.0213	(0.0805, 0.0832)	15.8534	0.4270	(15.8269, 15.8798)
	RF	0.0291	0.0166	(0.0280, 0.0301)	**0.7513**	0.0893	(0.7458, 0.7568)
	XGB	0.0362	0.0192	(0.0350, 0.0373)	1.7737	0.1130	(1.7667, 1.7807)
	Stacking	**0.0291**	0.0166	(0.0281, 0.0301)	0.8938	0.1477	(0.8846, 0.9030)
40%	KNN	0.0933	0.0275	(0.0916, 0.0950)	4.6314	0.3892	(4.6073, 4.6555)
	SVR	0.0962	0.0330	(0.0941, 0.0982)	11.8157	0.3085	(11.7966, 11.8349)
	DT	0.1298	0.0323	(0.1278, 0.1318)	15.9988	0.4616	(15.9702, 16.0274)
	RF	0.0758	0.0294	(0.0740, 0.0776)	**1.6014**	0.2076	(1.5885, 1.6143)
	XGB	0.0865	0.0330	(0.0845, 0.0886)	2.1502	0.1993	(2.1379, 2.1626)
	Stacking	**0.0754**	0.0294	(0.0736, 0.0773)	1.7986	0.2913	(1.7806, 1.8167)

Note: SD = Standard deviation; CI = 95% Confidence interval based on 1,000 simulation replications.

**Table 5:** Average MSIE and MAPIE with standard deviations (SD) and 95% confidence intervals (CI) for second DGP obtained from different imputation methods for *n* = 500.

Missing (%)	Method	MSIE	SD	95% CI	MAPIE	SD	95% CI
10%	KNN	0.3676	0.2314	(0.3533, 0.3820)	12.0459	1.5107	(11.9523, 12.1396)
	SVR	1.6701	0.7250	(1.6251, 1.7150)	23.8318	2.8544	(23.6549, 24.0087)
	DT	0.3260	0.1556	(0.3163, 0.3356)	13.8944	1.8032	(13.7827, 14.0062)
	RF	**0.2626**	0.1622	(0.2525, 0.2726)	**12.0768**	1.5849	(11.9785, 12.1750)
	XGB	0.4592	0.2511	(0.4436, 0.4748)	14.1779	1.7312	(14.0706, 14.2852)
	Stacking	0.2774	0.1024	(0.2711, 0.2838)	13.2928	1.6531	(13.1903, 13.3952)
20%	KNN	0.4367	0.2219	(0.4229, 0.4504)	12.4368	1.1236	(12.3671, 12.5064)
	SVR	1.9588	0.6688	(1.9174, 2.0003)	24.8043	2.2059	(24.6676, 24.9410)
	DT	0.3681	0.1576	(0.3584, 0.3779)	14.1224	1.3577	(14.0382, 14.2065)
	RF	0.3104	0.1595	(0.3005, 0.3202)	**12.4250**	1.1956	(12.3509, 12.4991)
	XGB	0.5056	0.2366	(0.4909, 0.5203)	14.4422	1.4117	(14.3547, 14.5297)
	Stacking	**0.2972**	0.1029	(0.2908, 0.3036)	13.4834	1.1979	(13.4092, 13.5576)
30%	KNN	0.5394	0.2332	(0.5249, 0.5538)	13.1578	1.1097	(13.0890, 13.2266)
	SVR	2.4496	0.7095	(2.4056, 2.4936)	26.8168	2.3359	(26.6720, 26.9616)
	DT	0.4434	0.1760	(0.4325, 0.4543)	14.6121	1.3126	(14.5308, 14.6935)
	RF	0.3812	0.1808	(0.3700, 0.3924)	**13.1222**	1.2949	(13.0419, 13.2025)
	XGB	0.5661	0.2309	(0.5517, 0.5804)	14.8520	1.5395	(14.7566, 14.9474)
	Stacking	**0.3197**	0.1128	(0.3127, 0.3266)	13.7921	1.0648	(13.7261, 13.8581)
40%	KNN	0.8329	0.2917	(0.8148, 0.8510)	15.4097	1.6932	(15.3048, 15.5147)
	SVR	3.7084	1.0165	(3.6454, 3.7714)	32.3931	4.1104	(32.1383, 32.6479)
	DT	0.5968	0.2053	(0.5841, 0.6096)	15.6320	1.3567	(15.5479, 15.7161)
	RF	0.5921	0.2549	(0.5763, 0.6079)	15.1827	2.1687	(15.0483, 15.3171)
	XGB	0.7242	0.2651	(0.7078, 0.7407)	15.9137	1.7681	(15.8041, 16.0233)
	Stacking	**0.4120**	0.1636	(0.4018, 0.4221)	**14.7793**	1.4016	(14.6924, 14.8661)

Note: SD = Standard deviation; CI = 95% Confidence interval based on 1,000 simulation replications.

**Table 6:** Average MSIE and MAPIE with standard deviations (SD) and 95% confidence intervals (CI) for second DGP obtained from different imputation methods for *n* = 1,000.

Missing (%)	Method	MSIE	SD	95% CI	MAPIE	SD	95% CI
10%	KNN	0.2913	0.1568	(0.2816, 0.3010)	**11.3772**	1.0283	(11.3135, 11.4410)
	SVR	1.7075	0.5583	(1.6729, 1.7421)	23.7636	1.9025	(23.6457, 23.8815)
	DT	0.3014	0.1088	(0.2946, 0.3081)	13.6676	1.2648	(13.5892, 13.7460)
	RF	**0.2316**	0.1223	(0.2240, 0.2392)	11.5435	1.1240	(11.4738, 11.6132)
	XGB	0.4314	0.1964	(0.4192, 0.4436)	13.8867	1.3158	(13.8051, 13.9682)
	Stacking	0.2371	0.0825	(0.2320, 0.2423)	12.4660	1.1444	(12.3951, 12.5369)
20%	KNN	0.3179	0.1226	(0.3103, 0.3255)	**11.6187**	0.7658	(11.5712, 11.6662)
	SVR	1.9569	0.4655	(1.9280, 1.9857)	24.8707	1.5787	(24.7729, 24.9686)
	DT	0.3110	0.0902	(0.3054, 0.3166)	13.8034	0.9616	(13.7438, 13.8630)
	RF	0.2490	0.0965	(0.2430, 0.2550)	11.7980	0.8533	(11.7451, 11.8508)
	XGB	0.4412	0.1517	(0.4318, 0.4506)	14.0721	1.1332	(14.0018, 14.1423)
	Stacking	**0.2378**	0.0585	(0.2341, 0.2414)	12.5472	0.8407	(12.4951, 12.5993)
30%	KNN	0.3872	0.1233	(0.3796, 0.3949)	**12.0936**	0.6983	(12.0503, 12.1369)
	SVR	2.4225	0.4880	(2.3922, 2.4527)	26.6416	1.6349	(26.5403, 26.7429)
	DT	0.3393	0.0874	(0.3338, 0.3447)	13.9763	0.8473	(13.9237, 14.0288)
	RF	0.2947	0.0974	(0.2887, 0.3008)	12.2537	0.7527	(12.2071, 12.3004)
	XGB	0.4763	0.1429	(0.4674, 0.4851)	14.2862	1.1388	(14.2156, 14.3568)
	Stacking	**0.2569**	0.0680	(0.2526, 0.2611)	12.8689	0.7290	(12.8237, 12.9141)
40%	KNN	0.5821	0.1638	(0.5720, 0.5923)	13.5321	0.9884	(13.4708, 13.5934)
	SVR	3.5899	0.6699	(3.5483, 3.6314)	31.8250	2.7175	(31.6566, 31.9935)
	DT	0.4481	0.1253	(0.4403, 0.4559)	14.6336	1.005	(14.5712, 14.6960)
	RF	0.4280	0.1332	(0.4197, 0.4362)	**13.5307**	1.1318	(13.4606, 13.6009)
	XGB	0.5936	0.1766	(0.5826, 0.6045)	15.1647	1.4906	(15.0723, 15.2571)
	Stacking	**0.3126**	0.0753	(0.3079, 0.3172)	13.6458	0.8399	(13.5937, 13.6978)

Note: SD = Standard deviation; CI = 95% Confidence interval based on 1,000 simulation replications.

**Table 7:** Average MSIE and MAPIE with standard deviations (SD) and 95% confidence intervals (CI) for second DGP obtained from different imputation methods for *n* = 5,000.

Missing (%)	Method	MSIE	SD	95% CI	MAPIE	SD	95% CI
10%	KNN	0.1801	0.0360	(0.1778, 0.1823)	10.9927	0.4452	(10.9646, 11.0196)
	SVR	1.6717	0.2535	(1.6560, 1.6874)	23.6532	0.8841	(23.5984, 23.7080)
	DT	0.2703	0.0461	(0.2675, 0.2732)	13.5044	0.5816	(13.4683, 13.5404)
	RF	**0.1740**	0.0314	(0.1720, 0.1759)	**10.6621**	0.4233	(10.6363, 10.6890)
	XGB	0.3729	0.0857	(0.3676, 0.3782)	13.4862	0.7894	(13.4373, 13.5352)
	Stacking	0.2032	0.0209	(0.2019, 0.2045)	12.3163	0.5001	(12.2853, 12.3473)
20%	KNN	0.2035	0.0261	(0.2026, 0.2043)	**10.7405**	0.3152	(10.7210, 10.7601)
	SVR	1.9362	0.2041	(1.9236, 1.9489)	24.7141	0.7118	(24.6700, 24.7582)
	DT	0.2802	0.0343	(0.2781, 0.2823)	13.5799	0.4642	(13.5512, 13.6087)
	RF	0.1897	0.0230	(0.1881, 0.1913)	11.0804	0.3360	(11.0596, 11.1013)
	XGB	0.3824	0.0764	(0.3777, 0.3871)	13.5870	0.8398	(13.5349, 13.6390)
	Stacking	**0.1812**	0.0142	(0.1798, 0.1826)	12.3305	0.3748	(12.3073, 12.3537)
30%	KNN	0.2142	0.0290	(0.2124, 0.2160)	**10.8952**	0.2667	(10.8786, 10.9117)
	SVR	2.4313	0.2257	(2.4173, 2.4452)	26.5939	0.7531	(26.5472, 26.6406)
	DT	0.3018	0.0323	(0.2998, 0.3038)	13.7476	0.3863	(13.7237, 13.7716)
	RF	0.2082	0.0252	(0.2073, 0.2091)	11.2285	0.2853	(11.2108, 11.2462)
	XGB	0.3972	0.0705	(0.3929, 0.4016)	13.7250	0.8538	(13.6721, 13.7779)
	Stacking	**0.1980**	0.0142	(0.1965, 0.1996)	12.3767	0.3051	(12.3578, 12.3956)
40%	KNN	0.2831	0.0451	(0.2803, 0.2859)	11.6489	0.3306	(11.6284, 11.6694)
	SVR	3.6013	0.3128	(3.5819, 3.6207)	31.7228	1.2552	(31.6450, 31.8006)
	DT	0.3310	0.0416	(0.3284, 0.3336)	13.9235	0.3794	(13.9000, 13.9470)
	RF	0.2466	0.0401	(0.2441, 0.2491)	**11.3426**	0.3029	(11.3238, 11.3614)
	XGB	0.4357	0.0853	(0.4304, 0.4410)	14.1688	1.0541	(14.1034, 14.2341)
	Stacking	**0.2279**	0.0209	(0.2266, 0.2292)	12.5548	0.3084	(12.5357, 12.5740)

Note: SD = Standard deviations; CI = 95% Confidence interval based on 1,000 simulation replications.

**Table 8:** Average MSIE and MAPIE with standard deviations (SD) and 95% confidence intervals (CI) for second DGP obtained from different imputation methods for *n* = 10,000.

Missing (%)	Method	MSIE	SD	95% CI	MAPIE	SD	95% CI
10%	KNN	0.1975	0.0177	(0.1968, 0.1981)	**10.5567**	0.3072	(10.5377, 10.5758)
	SVR	1.6846	0.1732	(1.6738, 1.6953)	23.7058	0.6130	(23.6678, 23.7438)
	DT	0.2669	0.0295	(0.2650, 0.2687)	13.5033	0.4532	(13.4752, 13.5314)
	RF	0.1637	0.0162	(0.1627, 0.1647)	10.8794	0.3177	(10.8597, 10.8991)
	XGB	0.3652	0.0662	(0.3611, 0.3693)	13.3978	0.6921	(13.3549, 13.4407)
	Stacking	**0.1618**	0.0108	(0.1607, 0.1629)	12.2614	0.3374	(12.2405, 12.2824)
20%	KNN	0.1988	0.0154	(0.1983, 0.1994)	**10.5960**	0.2539	(10.5824, 10.6095)
	SVR	1.9446	0.1454	(1.9356, 1.9537)	24.7191	0.5000	(24.6881, 24.7501)
	DT	0.2827	0.0277	(0.2810, 0.2844)	13.6278	0.3499	(13.6061, 13.6495)
	RF	0.1689	0.0143	(0.1680, 0.1698)	12.2736	0.2539	(12.257,8 12.2893)
	XGB	0.3690	0.0629	(0.3651, 0.3729)	13.4215	0.7421	(13.3755, 13.4675)
	Stacking	**0.1686**	0.0088	(0.1676, 0.1695)	10.9300	0.2349	(10.9155, 10.9446)
30%	KNN	0.2017	0.0170	(0.2011, 0.2022)	12.2968	0.0784	(12.283,4 12.3102)
	SVR	2.4215	0.1553	(2.4119, 2.4311)	26.5640	0.4328	(26.5321, 26.5960)
	DT	0.2994	0.0247	(0.2979, 0.3009)	13.7215	0.2638	(13.7040, 13.7391)
	RF	0.1819	0.0152	(0.1809, 0.1830)	**10.6727**	0.0667	(10.6614, 10.6840)
	XGB	0.3853	0.0664	(0.3812, 0.3894)	13.6401	0.0851	(13.5884, 13.6918)
	Stacking	**0.1782**	0.0092	(0.1772, 0.1791)	11.0104	0.0994	(10.9986, 11.0222)
40%	KNN	0.2234	0.0234	(0.2219, 0.2248)	10.9438	0.1830	(10.9325, 10.9551)
	SVR	3.5846	0.2129	(3.5714, 3.5978)	31.6673	0.8579	(31.6141, 31.7204)
	DT	0.3202	0.0243	(0.3186, 0.3217)	13.8476	0.2831	(13.8301, 13.8652)
	RF	0.2147	0.0215	(0.2139, 0.2154)	**11.2797**	0.2031	(11.2671, 11.2923)
	XGB	0.4068	0.0721	(0.4023, 0.4112)	13.9967	1.0431	(13.9320, 14.0613)
	Stacking	**0.2081**	0.0120	(0.2068, 0.2094)	12.4142	0.2136	(12.4009, 12.4274)

Note: SD = Standard deviations; CI = 95% Confidence interval based on 1,000 simulation replications.

**Table 9:** Average MSIE and MAPIE with standard deviations (SD) and 95% confidence intervals (CI) for third DGP obtained from different imputation methods for *n* = 500.

Missing (%)	Method	MSIE	SD	95% CI	MAPIE	SD	95% CI
10%	KNN	18.3520	43.2389	(15.7621,21.0320)	**20.4433**	47.8066	(17.4802,23.4064)
	SVR	22.1688	43.8839	(19.4488,24.8887)	38.7250	48.8618	(35.6965,41.7535)
	DT	17.8632	41.6670	(15.2807,20.4458)	58.2865	157.1814	(48.5443, 68.0287)
	RF	16.1010	36.5097	(13.8381,18.3639)	79.1771	567.2517	(44.0185,114.3358)
	XGB	16.6948	41.1086	(14.1468,19.2427)	20.9070	36.0023	(18.6756,23.1385)
	Stacking	**15.0659**	38.7820	(12.6622,17.4696)	30.0663	114.8581	(22.9473,37.1852)
20%	KNN	18.1443	22.0484	(16.7777,19.5109)	**29.8380**	235.7973	(15.2231,44.4528)
	SVR	21.3872	22.3637	(20.0010,22.7733)	58.2587	582.4808	(22.1562,94.3613)
	DT	18.0934	21.4087	(16.7664,19.4203)	82.6251	865.5305	(28.9790,136.2712)
	RF	16.6026	19.4635	(15.3962,17.8089)	90.9442	962.2692	(31.3021,150.5862)
	XGB	16.8073	21.1141	(15.4986,18.1159)	31.4706	256.0870	(15.5982,47.3430)
	Stacking	**15.2873**	20.1414	(14.0389,16.5356)	35.2864	214.1994	(22.0102,48.5626)
30%	KNN	22.3955	22.5984	(20.9948, 23.7962)	33.1851	171.1482	(22.5772,43.7930)
	SVR	24.2726	22.4626	(22.8803, 25.6648)	44.0634	52.8640	(40.7868,47.3399)
	DT	22.4062	21.8825	(21.0499, 23.7625)	65.1593	264.0220	(48.7950,81.5235)
	RF	21.5532	20.5114	(20.2819, 22.8245)	62.1797	85.8734	(56.8573,67.5022)
	XGB	21.4295	21.9985	(20.0661, 22.7930)	**33.0239**	168.5740	(22.5756,43.4723)
	Stacking	**19.9999**	20.9933	(18.6987, 21.3011)	35.1651	167.6307	(24.7752,45.5549)
40%	KNN	27.2283	21.1768	(25.9157, 28.5409)	40.9371	65.6883	(36.8657,45.0085)
	SVR	26.3051	20.2965	(25.0471, 27.5630)	52.3428	143.2994	(43.4610,61.2246)
	DT	27.7595	21.1114	(26.4510, 29.0680)	80.0990	479.3851	(50.3864,109.8116)
	RF	28.0355	20.2912	(26.7778, 29.2931)	72.9789	332.4083	(52.3760,93.5818)
	XGB	26.5820	20.9091	(25.2861, 27.8780)	**38.6821**	55.7370	(35.2275,42.1367)
	Stacking	**25.9101**	20.5340	(24.6374, 27.1828)	42.4483	173.6934	(31.6826,53.2139)

Note: SD = Standard deviations; CI = 95% Confidence interval based on 1,000 simulation replications.

**Table 10:** Average MSIE and MAPIE with standard deviations (SD) and 95% confidence intervals (CI) for third DGP obtained from different imputation methods for *n* = 1000.

Missing (%)	Method	MSIE	SD	95% CI	MAPIE	SD	95% CI
10%	KNN	12.4771	18.8390	(11.3095, 13.6448)	**18.3291**	47.3871	(15.3920,21.2662)
	SVR	18.1805	21.3196	(16.8591,19.5019)	38.4124	53.3059	(35.1084,41.7163)
	DT	12.3356	17.7184	(11.2374,13.4338)	56.3842	180.3158	(45.2081,67.5602)
	RF	11.6392	15.3757	(10.6862,12.5922)	77.4326	386.2329	(53.4936,101.3715)
	XGB	10.9666	17.2433	(9.8978,12.0353)	19.2368	43.9608	(16.5121,21.9615)
	Stacking	**9.7667**	15.8278	(8.7857,10.7477)	30.1774	120.1616	(22.7297,37.6251)
20%	KNN	14.3660	15.8109	(13.3860,15.3460)	**29.4732**	365.2306	(6.8360, 52.1104)
	SVR	19.5037	17.2943	(18.4317,20.5756)	39.6624	100.9687	(33.4043,45.9205)
	DT	14.4701	14.9715	(13.5422,15.3981)	76.0474	687.3206	(33.4468,118.6480)
	RF	14.1786	13.9452	(13.3142,15.0429)	95.7302	848.6574	(43.1299,148.3306)
	XGB	13.2777	14.9466	(12.3513,14.2041)	31.5478	410.1349	(6.1274,56.9683)
	Stacking	**12.0232**	14.1658	(11.1452,12.9012)	42.9038	524.7327	(10.3806,75.4271)
30%	KNN	18.7947	17.1228	(17.7334,19.8560)	**24.7624**	76.6789	(20.0098,29.5150)
	SVR	23.0170	17.6784	(21.9213,24.1127)	44.0275	121.9744	(36.4675,51.5876)
	DT	19.1144	16.6610	(18.0817,20.1470)	77.5171	419.6440	(51.5073,103.5269)
	RF	19.9677	15.6756	(18.9962,20.9393)	99.2158	621.1005	(60.7196,137.7120)
	XGB	17.9607	21.9985	(16.9404,18.9810)	25.3092	83.9361	(20.1068,30.5117)
	Stacking	**16.9345**	15.9022	(15.9488,17.9201)	38.7532	220.8042	(25.0676,52.4388)
40%	KNN	24.0391	15.8390	(23.0574,25.0208)	40.9131	296.6931	(22.5239,59.3023)
	SVR	25.2090	15.2307	(24.2650,26.1530)	43.4853	55.4046	(40.0513,46.9194)
	DT	24.7892	15.7489	(23.8131,25.7653)	111.8118	1344.6765	(28.4679,195.1557)
	RF	28.1212	15.7685	(27.1438,29.0985)	126.8925	1257.0688	(48.9786,204.8065)
	XGB	23.6331	15.7135	(22.6592,24.6070)	38.3272	243.6495	(23.2256,53.4287)
	Stacking	**23.4637**	15.5820	(22.4979,24.4294)	**35.3685**	60.7717	(31.6018,39.1351)

Note: SD = Standard deviations; CI = 95% Confidence interval based on 1,000 simulation replications.

**Table 11:** Average MSIE and MAPIE with standard deviations (SD) and 95% confidence intervals (CI) for third DGP obtained from different imputation methods for *n* = 5000.

Missing (%)	Method	MSIE	SD	95% CI	MAPIE	SD	95% CI
10%	KNN	6.8545	9.9849	(6.2356, 7.4734)	**14.3254**	33.5380	(12.2467, 16.4041)
	SVR	14.4384	12.5906	(13.6580, 15.2188)	43.5862	90.9892	(37.9466, 49.2257)
	DT	7.6000	9.2247	(7.0283, 8.1718)	52.4470	78.6192	(47.5741, 57.3198)
	RF	9.5118	9.7423	(8.9080, 10.1157)	91.5457	299.1807	(73.0023, 110.0892)
	XGB	6.4023	9.4446	(5.8169, 6.9877)	15.3115	35.5979	(13.1051, 17.5179)
	Stacking	**5.9891**	9.0828	(5.4262 6.5521)	26.4673	50.0954	(23.3624, 29.5722)
20%	KNN	7.8965	8.7229	(7.3558, 8.4371)	**13.6264**	14.5015	(12.7276, 14.5252)
	SVR	15.6502	10.5430	(14.9967, 16.3036)	40.9545	48.3860	(37.9555, 43.9535)
	DT	8.7244	8.6001	(8.1914, 9.2574)	53.3082	74.0570	(48.7181, 57.8983)
	RF	11.1107	8.4632	(10.5861, 11.6352)	84.2663	168.0202	(73.8523, 94.6803)
	XGB	7.4864	8.3964	(6.9660, 8.0069)	14.4937	14.5383	(13.5926, 15.3947)
	Stacking	**7.0660**	8.1984	(6.5579, 7.5741)	23.8919	14.7855	(22.9754, 24.8083)
30%	KNN	9.2376	5.5250	(8.8952, 9.5801)	**14.4935**	21.9517	(13.1330, 15.8541)
	SVR	16.8038	6.6663	(16.3906, 17.2169)	44.3780	45.8293	(41.5374, 47.2185)
	DT	10.1568	5.5490	(9.8128, 10.5007)	53.2421	33.3633	(51.1743, 55.3100)
	RF	13.6757	5.7731	(13.3179, 14.0335)	88.1513	228.1394	(74.0111, 102.2916)
	XGB	8.9679	5.4451	(8.6304, 9.3054)	15.4119	21.9740	(14.0499, 16.7738)
	Stacking	**8.6366**	5.3630	(8.3042, 8.9690)	25.3878	26.2964	(23.7580, 27.0177)
40%	KNN	14.1668	6.6807	(13.7238, 14.5520)	**18.8025**	18.1343	(17.6786, 19.9265)
	SVR	20.3776	7.1946	(19.9317, 20.8235)	60.5421	131.4968	(52.3919, 68.6924)
	DT	15.0885	6.6737	(14.6749, 15.5022)	69.8215	331.6766	(49.2640, 90.3790)
	RF	21.4360	7.2368	(20.9875, 21.8846)	95.6605	337.2921	(74.7549, 116.5661)
	XGB	14.2048	6.6695	(13.7914, 14.6181)	20.4275	49.5563	(17.3560, 23.4990)
	Stacking	**14.1379**	6.6512	(13.7545, 14.5790)	32.4791	163.8629	(22.3228, 42.6354)

Note: SD = Standard deviations; CI = 95% Confidence interval based on 1,000 simulation replications.

**Table 12:** Average MSIE and MAPIE with standard deviations (SD) and 95% confidence intervals (CI) for third DGP obtained from different imputation methods for *n* = 10,000.

Missing (%)	Method	MSIE	SD	95% CI	MAPIE	SD	95% CI
10%	KNN	4.8381	7.5460	(4.3704, 5.3058)	**15.9467**	64.0954	(11.9740, 19.9194)
	SVR	12.5054	9.5029	(11.9164, 13.0944)	55.4162	237.5534	(40.6925, 70.1399)
	DT	5.8581	7.0490	(5.4224, 6.2938)	56.6349	167.0151	(46.2832, 66.9866)
	RF	8.6552	7.8151	(8.1708, 9.1396)	113.0458	557.7614	(78.4754, 147.6162)
	XGB	4.5799	7.1552	(4.1364, 5.0234)	18.3631	93.1739	(12.5881, 24.1381)
	Stacking	**4.4604**	7.0254	(4.0237, 4.8971)	33.9549	191.2355	(22.1020, 45.8078)
20%	KNN	5.3622	4.0043	(5.1140 5.6104)	**17.1835**	135.9465	(8.7575, 25.6096)
	SVR	13.3059	5.6054	(12.9585 13.6534)	52.1141	200.3624	(39.6955, 64.5327)
	DT	6.3880	3.9112	(6.1456 6.6304)	56.5381	174.4483	(45.7257, 67.3505)
	RF	9.9974	4.3325	(9.7288 10.2659)	113.6722	723.5002	(68.8292, 158.5152)
	XGB	5.2120	3.8699	(4.9722 5.4519)	18.4635	143.1471	(9.5912, 27.3359)
	Stacking	**5.0806**	3.7963	(4.8453 5.3159)	25.2806	23.5945	(23.8182, 26.7430)
30%	KNN	6.8082	4.2422	(6.5453, 7.0712)	**15.4365**	50.3423	(12.3163, 18.5568)
	SVR	15.1290	5.4290	(14.7926, 15.4655)	58.8995	192.1523	(46.9897, 70.8092)
	DT	7.8922	4.2391	(7.6294, 8.1549)	64.4586	273.6829	(47.4956, 81.4216)
	RF	12.6434	4.6552	(12.3549, 12.9319)	114.0050	438.9698	(86.7974, 141.2127)
	XGB	6.6215	4.1851	(6.3621, 6.8809)	17.1308	67.3363	(12.9572, 21.3043)
	Stacking	**6.5848**	4.1605	(6.3269, 6.8426)	31.0996	153.2986	(21.5980, 40.6011)
40%	KNN	10.0275	4.2470	(9.7643, 10.2907)	**15.3004**	13.1201	(14.4872, 16.1136)
	SVR	18.0039	5.0467	(17.6911, 18.3167)	62.6541	96.1326	(56.6958, 68.6125)
	DT	11.1109	4.3129	(10.8436, 11.3782)	62.3652	93.8395	(56.5490, 68.1814)
	RF	18.2481	4.9042	(17.9442, 18.5521)	97.5629	250.7643	(82.0204, 113.1055)
	XGB	10.1401	4.2737	(9.7207, 10.2465)	16.1468	18.9530	(14.9721, 17.3215)
	Stacking	**9.9836**	4.2419	(9.8752, 10.4050)	26.6721	33.2362	(24.6121, 28.7321)

Note: SD = Standard deviations; CI = 95% Confidence interval based on 1,000 simulation replications.

**Table 13:** Average MSIE and MAPIE with standard deviations (SD) and 95% confidence intervals (CI) for fourth DGP obtained from different imputation methods for *n* = 500.

Missing (%)	Method	MSIE	SD	95% CI	MAPIE	SD	95% CI
10%	KNN	1.4185	0.5041	(1.3888, 1.4481)	36.3814	5.5763	(36.0357, 36.7270)
	SVR	1.4313	0.6402	(1.3917, 1.4710)	**35.9137**	6.1378	(35.5333, 36.2941)
	DT	1.4660	0.5216	(1.4337, 1.4983)	38.3443	6.1947	(37.9603, 38.7282)
	RF	1.4127	0.5134	(1.3809, 1.4445)	37.4735	5.9608	(37.1041, 37.8430)
	XGB	1.5432	0.5295	(1.5104, 1.5760)	39.7064	6.4287	(39.3080, 40.1049)
	Stacking	**1.4040**	0.4779	(1.3728, 1.4353)	37.7274	6.0322	(37.3535, 38.1013)
20%	KNN	1.4791	0.3945	(1.4546, 1.5036)	36.8777	4.0548	(36.6264, 37.1290)
	SVR	1.4951	0.5077	(1.4636, 1.5266)	**36.3995**	4.5178	(36.1195, 36.6796)
	DT	1.5041	0.3811	(1.4807, 1.5274)	39.1918	4.5894	(38.9073, 39.4762)
	RF	1.4530	0.3976	(1.4284, 1.4776)	38.2450	4.4101	(37.9716, 38.5183)
	XGB	1.6035	0.4043	(1.5784, 1.6286)	40.8737	4.8725	(40.5717, 41.1757)
	Stacking	**1.4465**	0.3767	(1.4229, 1.4701)	38.3474	4.4754	(38.0700, 38.6248)
30%	KNN	1.6806	0.4092	(1.6552, 1.7060)	38.4955	3.7404	(38.2637, 38.7274)
	SVR	1.7064	0.5170	(1.6744, 1.7385)	**37.6659**	4.0081	(37.4175, 37.9143)
	DT	1.6561	0.3914	(1.6319, 1.6804)	40.3714	3.9120	(40.1290, 40.6139)
	RF	1.5946	0.3982	(1.5699, 1.6193)	39.2573	3.8831	(39.0167, 39.4980)
	XGB	1.7869	0.4029	(1.7619, 1.8118)	42.4364	4.4070	(42.1632, 42.7095)
	Stacking	**1.5917**	0.3822	(1.5680, 1.6154)	39.3300	3.9239	(39.0867, 39.5732)
40%	KNN	2.2704	0.6366	(2.2310, 2.3099)	44.4013	5.4497	(44.0635, 44.7391)
	SVR	2.1803	0.6062	(2.1428, 2.2179)	**41.1390**	4.6514	(40.8507, 41.4273)
	DT	2.1271	0.5972	(2.0901, 2.1641)	44.3010	5.1639	(43.9810, 44.6211)
	RF	2.0629	0.5960	(2.0160, 2.0898)	42.4881	4.7733	(42.1922, 42.7839)
	XGB	2.3372	0.6271	(2.2984, 2.3761)	46.8739	5.1787	(46.5530, 47.1949)
	Stacking	**2.0519**	0.6012	(2.0247, 2.0992)	42.8521	4.8294	(42.5528, 43.1514)

Note: SD = Standard deviations; CI = 95% Confidence interval based on 1,000 simulation replications.

**Table 14:** Average MSIE and MAPIE with standard deviations (SD) and 95% confidence intervals (CI) for fourth DGP obtained from different imputation methods for *n* = 1000.

Missing (%)	Method	MSIE	SD	95% CI	MAPIE	SD	95% CI
10%	KNN	1.2699	0.2801	(1.2531, 1.2868)	35.2435	4.0113	(34.9949, 35.4922)
	SVR	1.2646	0.3673	(1.2418, 1.2873)	**34.7493**	4.1316	(34.4933, 35.0054)
	DT	1.3248	0.2994	(1.3062, 1.3433)	37.2207	4.2667	(36.9563, 37.4852)
	RF	1.3072	0.3197	(1.2874, 1.3270)	36.8369	4.2719	(36.5721, 37.1016)
	XGB	1.3182	0.2811	(1.3008, 1.3356)	37.3823	4.3981	(37.1098, 37.6549)
	Stacking	**1.2596**	0.2719	(1.2422, 1.2769)	36.5714	4.2977	(36.3050, 36.8377)
20%	KNN	1.3375	0.2155	(1.3242, 1.3509)	35.8951	2.9548	(35.7120, 36.0783)
	SVR	1.3485	0.3052	(1.3296, 1.3674)	**35.3743**	3.1450	(35.1794, 35.5692)
	DT	1.3790	0.2280	(1.3649, 1.3931)	38.0176	3.2244	(37.8178, 38.2175)
	RF	1.3593	0.2464	(1.3440, 1.3746)	37.4264	3.1670	(37.2301, 37.6227)
	XGB	1.3934	0.2211	(1.3797, 1.4071)	38.2077	3.2449	(38.0066, 38.4088)
	Stacking	**1.3183**	0.2143	(1.3050, 1.3316)	37.1856	3.1811	(36.9884, 37.3828)
30%	KNN	1.8081	0.3288	(1.7878, 1.8285)	40.0400	3.0053	(39.8537, 40.2263)
	SVR	1.8574	0.3787	(1.8339, 1.8809)	**38.7129**	3.2217	(38.5132, 38.9126)
	DT	1.7378	0.3254	(1.7177, 1.7580)	40.7573	2.9651	(40.5735, 40.9411)
	RF	1.7239	0.3230	(1.7039, 1.7439)	40.2159	3.0313	(40.0280, 40.4038)
	XGB	1.8420	0.3370	(1.8211, 1.8628)	42.2174	3.1673	(42.0211, 42.4137)
	Stacking	**1.6845**	0.3140	(1.6651, 1.7040)	39.9710	2.9619	(39.7874, 40.1546)
40%	KNN	1.1241	0.0837	(1.1189, 1.1293)	34.6459	1.9200	(34.5269, 34.7649)
	SVR	1.0873	0.1147	(1.0802, 1.0944)	**33.6486**	1.8739	(33.5324, 33.7647)
	DT	1.1843	0.1033	(1.1779, 1.1907)	35.7593	1.9487	(35.6385, 35.8801)
	RF	1.2246	0.1221	(1.2171, 1.2322)	36.2399	1.8451	(36.1255, 36.3542)
	XGB	1.1048	0.0854	(1.0995, 1.1101)	34.6412	1.8488	(34.5266, 34.7558)
	Stacking	**1.0987**	0.0861	(1.0933, 1.1040)	34.5966	1.8857	(34.4797, 34.7135)

Note: SD = Standard deviations; CI = 95% Confidence interval based on 1,000 simulation replications.

**Table 15:** Average MSIE and MAPIE with standard deviations (SD) and 95% confidence intervals (CI) for fourth DGP obtained from different imputation methods for *n* = 5000.

Missing (%)	Method	MSIE	SD	95% CI	MAPIE	SD	95% CI
10%	KNN	1.1241	0.0854	(1.1189, 1.1293)	34.6459	1.9200	(34.5269, 34.7649)
	SVR	1.0987	0.1147	(1.0933, 1.1040)	**33.6486**	1.8739	(33.5324, 33.7647)
	DT	1.1843	0.1033	(1.1779, 1.1907)	35.7593	1.9487	(35.6385, 35.8801)
	RF	1.2246	0.1221	(1.2171, 1.2322)	36.2399	1.8451	(36.1255, 36.3542)
	XGB	1.1048	0.0861	(1.0995, 1.1101)	34.6412	1.8488	(34.5266, 34.7558)
	Stacking	**1.0873**	0.0837	(1.0802, 1.0944)	34.5966	1.8857	(34.4797, 34.7135)
20%	KNN	1.1414	0.0659	(1.1374, 1.1454)	34.6599	1.4034	(34.5730, 34.7469)
	SVR	1.1182	0.0887	(1.1141, 1.1223)	**33.7533**	1.4035	(33.6663, 33.8403)
	DT	1.2046	0.0827	(1.1995, 1.2097)	35.8246	1.4463	(35.7350, 35.9143)
	RF	1.2438	0.0940	(1.2380, 1.2497)	36.3559	1.4156	(36.2682, 36.4437)
	XGB	1.1258	0.0664	(1.1218, 1.1299)	34.8305	1.3903	(34.7444, 34.9167)
	Stacking	**1.1127**	0.0650	(1.1072, 1.1182)	34.7715	1.4203	(34.6834, 34.8595)
30%	KNN	1.1774	0.0608	(1.1737, 1.1812)	34.8168	1.2032	(34.7422, 34.8914)
	SVR	1.1688	0.0878	(1.1633, 1.1742)	**34.0457**	1.2382	(33.9689, 34.1224)
	DT	1.2454	0.0780	(1.2405, 1.2502)	36.1791	1.2993	(36.0986, 36.2597)
	RF	1.2877	0.0862	(1.2823, 1.2930)	36.8604	1.3172	(36.7788, 36.9421)
	XGB	1.1656	0.0604	(1.1618, 1.1693)	35.2562	1.2318	(35.1799, 35.3326)
	Stacking	**1.1555**	0.0601	(1.1518, 1.1592)	35.1362	1.2629	(35.0579, 35.2145)
40%	KNN	1.2812	0.0825	(1.2761, 1.2863)	35.5443	1.1568	(35.4726, 35.6160)
	SVR	1.3242	0.1169	(1.3169, 1.3314)	**34.9874**	1.2923	(34.9073, 35.0675)
	DT	1.3369	0.0982	(1.3308, 1.3429)	37.0888	1.3506	(37.0051, 37.1725)
	RF	1.3800	0.0986	(1.3739, 1.3861)	37.9583	1.3977	(37.8717, 38.0449)
	XGB	1.2658	0.0877	(1.2603, 1.2712)	36.2707	1.2881	(36.1909, 36.3506)
	Stacking	**1.2423**	0.0818	(1.2372, 1.2475)	36.0429	1.2764	(35.9638, 36.1220)

Note: SD = Standard deviations; CI = 95% Confidence interval based on 1,000 simulation replications.

**Table 16:** Average MSIE and MAPIE with standard deviations (SD) and 95% confidence intervals (CI) for fourth DGP obtained from different imputation methods for *n* = 10,000.

Missing (%)	Method	MSIE	SD	95% CI	MAPIE	SD	95% CI
10%	KNN	0.5284	0.5504	(0.4943, 0.5625)	16.6218	17.2993	(15.5496, 17.6940)
	SVR	0.5094	0.5306	(0.4765, 0.5423)	**16.0794**	16.7377	(15.0419, 17.1168)
	DT	0.5565	0.5803	(0.5205, 0.5925)	17.0784	17.7756	(15.9767, 18.1802)
	RF	0.5848	0.6106	(0.5469, 0.6226)	17.4273	18.1363	(16.3032, 18.5514)
	XGB	0.5110	0.5322	(0.4780, 0.5439)	16.4050	17.0733	(15.3468, 17.4632)
	Stacking	**0.5048**	0.5267	(0.4721, 0.5374)	16.4025	17.0731	(15.3443, 17.4607)
20%	KNN	0.2234	0.0234	(0.2219, 0.2248)	**10.9438**	0.1830	(10.9325, 10.9551)
	SVR	0.5846	0.2129	(0.5714, 0.5978)	13.6673	0.8579	(13.6141, 13.7204)
	DT	0.3202	0.0243	(0.3186, 0.3217)	13.8476	0.2831	(13.8301, 13.8652)
	RF	0.2147	0.0120	(0.2139, 0.2154)	11.2797	0.2031	(11.2671, 11.2923)
	XGB	0.4068	0.0721	(0.4023, 0.4112)	13.9967	1.0431	(13.9320, 14.0613)
	Stacking	**0.2081**	0.0215	(0.2068, 0.2094)	12.4142	0.2136	(12.4009, 12.4274)
30%	KNN	0.7667	0.5753	(0.7310, 0.8024)	22.4189	16.7975	(21.3777, 23.4600)
	SVR	0.8250	0.6200	(0.7866, 0.8634)	24.0500	17.9094	(22.9390, 25.1610)
	DT	0.7890	0.6013	(0.7528, 0.8272)	23.2037	17.1996	(22.1330, 24.2670)
	RF	0.6900	0.5196	(0.6578, 0.7222)	**20.4926**	15.2041	(19.5570, 21.4430)
	XGB	0.7350	0.5547	(0.7009, 0.7691)	22.0719	16.3981	(21.0830, 23.1170)
	Stacking	**0.6523**	0.5074	(0.6190, 0.6810)	21.0801	15.7165	(20.0260, 21.9740)
40%	KNN	0.9090	0.4474	(0.8813, 0.9367)	27.9485	13.7400	(27.0969, 28.8001)
	SVR	0.8860	0.4374	(0.8589, 0.9131)	**27.1622**	13.3550	(26.3344, 27.9899)
	DT	0.9628	0.4754	(0.9334, 0.9923)	28.7815	14.1538	(27.9042, 29.6587)
	RF	1.0143	0.5009	(0.9832, 1.0453)	29.5686	14.5408	(28.6673, 30.4698)
	XGB	0.8865	0.4364	(0.8594, 0.9136)	27.8141	13.6723	(26.9667, 28.6615)
	Stacking	**0.8726**	0.4346	(0.8557, 0.9096)	27.7490	13.6419	(26.9034, 28.5945)

Note: SD = Standard deviations; CI = 95% Confidence interval based on 1,000 simulation replications.

### Application to real-life data

To further evaluate the effectiveness of the proposed imputation framework, two real-world datasets were utilized. The application of the proposed and benchmark imputation techniques to real datasets provides valuable insight into their practical implications and predictive performance in the presence of missing data. Moreover, the findings further demonstrate the robustness, applicability, and generalizability of the proposed methodology in real-data domains.

For the assessment of the proposed methodology on real datasets, variables from the “Wine Quality” dataset available at https://doi.org/10.24432/C56S3T were used to validate the proposed method. In this dataset, alcohol, volatile acidity, and sulphates were selected as covariates, whereas wine quality was considered as the target study variable. Similarly, variables from the “Real-time Election Results: Portugal 2019” dataset available at https://doi.org/10.24432/C5NS5M were considered. Specifically, five variables namely total number of votes, vote percentage, Hondt allocation index, valid votes percentage, and voter turnout percentage were selected as covariates, while the final mandate count was treated as the target study variable. All selected variables were standardized to a common scale to ensure equal contribution of each variable during the imputation process. Fig 3 and Fig 4 present the performance results obtained from the two real-life datasets. The findings are consistent with those observed in the simulation studies, indicating that the results from the real-life applications align closely with the simulation-based outcomes.

**Fig 3:**
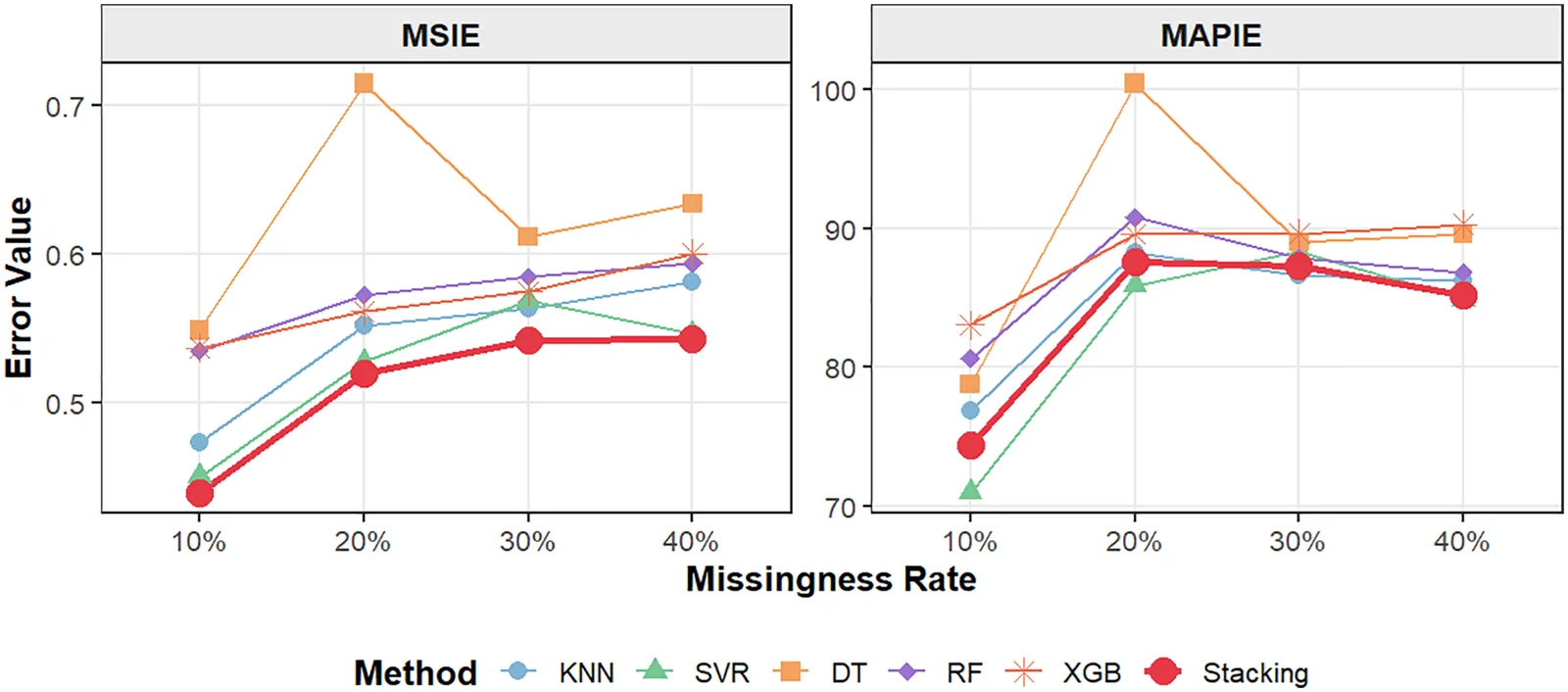
MSIE and MAPIE across missingness rates for the Wine Quality dataset.

**Fig 4:**
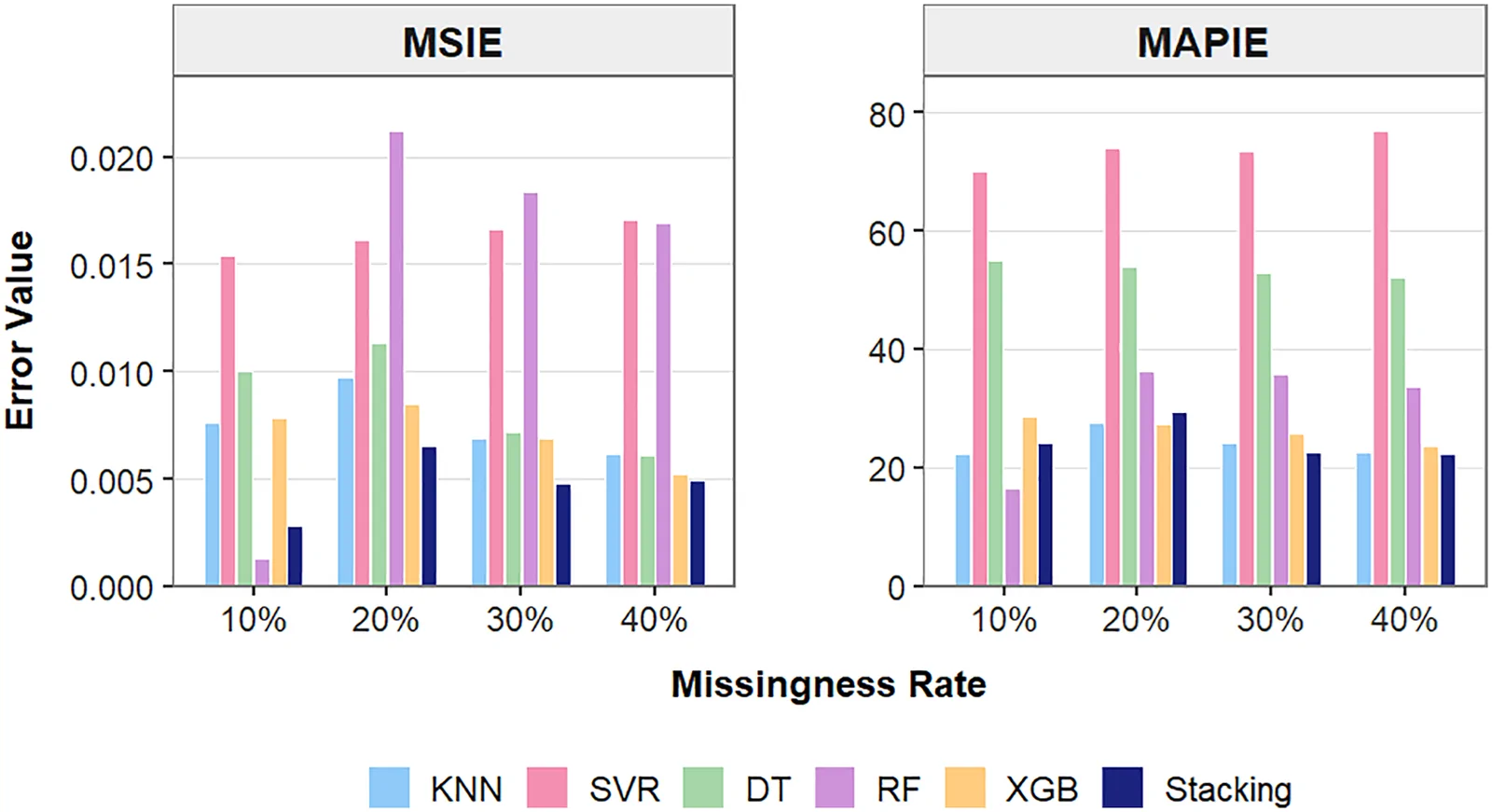
MSIE and MAPIE across missingness rates for the Real-time Election Results: Portugal 2019 dataset.

## Conclusion

An imputation approach for handling missing data was developed for application to regression tasks. Specifically, a stacking ensemble-based approach, referred to as layered stacking, was proposed. Standard machine learning methods were integrated into a layered architecture, serving as an effective approach to the imputation of continuous missing data. The performance of the proposed method was evaluated through a series of simulated experiments, conducted using four different sample sizes with an MAR mechanism applied with four levels of missingness from 10 to 40%. The results were compared against five machine learning imputation methods: k-nearest neighbors, support vector regression, decision tree, random forest and extreme gradient boosting. The simulation results demonstrated that the proposed method generally performed more favorably than the baseline models across most sample sizes and missing proportions. To further validate the robustness and practical applicability of the proposed method, two publicly available real-world datasets from the UCI Machine Learning Repository were also used. The findings obtained from these real-data applications were consistent with the results of the simulation studies, thereby further supporting the reliability and generalizability of the proposed approach.

## Limitations and future recommendations

Several important challenges remain unaddressed in the domain of missing value imputation, presenting opportunities for future research. One key issue is the difficulty of developing a universally effective imputation method that performs reliably across all types of missing data mechanisms, whether statistical or machine learning-based. This remains a critical objective for future studies. Additionally, missing data can significantly impact the accuracy of regression models and reduce the effectiveness of downstream processes. In light of these challenges, continued exploration of advanced machine learning approaches such as the proposed layered stacking method holds considerable potential. Future research should further investigate how ensemble-based strategies can be adapted or extended to enhance generalizability, especially under different mechanisms, i.e., MCAR and MNAR, with varied missingness conditions and data distributions.
